# Hidden Psychiatric Morbidities and General Health Status Among Men Who have Sex with Men and Other Clients of a Sexually Transmitted Disease Clinic of Kolkata: A Comparative Study

**DOI:** 10.4103/0970-0218.62566

**Published:** 2010-01

**Authors:** Soumya Deb, Sinjita Dutta, Aparajita Dasgupta, Sarbojit Roy

**Affiliations:** Department of Community Medicine, IPGMER, SSKM Hospital, Kolkata - 700 073, India; 1 Department of Preventive and Social Medicine (PSM), All India Institute of Hygiene and Public Health, 110, C.R. Avenue, Kolkata - 700 073, India

## Introduction

Next to South Africa, India has the second largest number of persons living with HIV/AIDS in the world. According to the data on the National AIDS Control Organization till 7^th^ July, 2008, adult HIV prevalence in India is approximately 0.36%, amounting to between 2 and 3.1 million people. It is also estimated that 85% of HIV transmission is sexual. Though the contribution of MSM to the HIV/AIDS epidemic in India was officially set at 1% in 2001,(1) it is felt by the researchers that this Figure underestimates the impact of unsafe sexual practice of the MSM on the epidemic of HIV/AIDS in India, especially since global estimates suggest that 5 to 10% of HIV prevalence is attributable to sexual transmission between men.(2)

The frameworks of male-to-male sex are substantially divergent and inclusive. It includes normative males (*panthis*) who desire to penetrate as the only signifier, feminized males (*kothis*) who desire to be penetrated, males who have partners of both sex (*duplis*) along with *hijras*, who desire to be penetrated by other males, and adolescent and other males who desire to experiment for fun. Men Who Have Sex with Men (MSM) are a unique group of people who live in a world of utopia. They feel and believe they will face hardships in society once their identity is disclosed but this does not deter them from dressing and behaving like the opposite sex in public life or fighting for their rights. Not to forget they are humans, many a times they are taunted by common man in the street. The more they are treated like social outcaste, the more they will try to conceal their feelings and perception, the net result this population will remain on the fringes of the society. Ultimately, they will remain unaware about them being a very high risk vulnerable group in the recent upsurge in HIV/AIDS transmission and their ignorance will help in spreading of the disease further in the community. No wonder, the inner conflicts in a MSM grows day by day and it takes a toll on their social life and hurts their mind. As a result, psychiatric morbidities like depression, anxiety, psychotic disorders and even suicidal tendencies are common in this group.

With this in backdrop, the researchers decided to conduct a study in a MSM clinic of central Kolkata. The clinic is run under the auspices of an NGO, Manas Bangla, which provides care and treatment to the MSM in eastern India especially West Bengal.

## Materials and Methods

A descriptive, cross-sectional study was conducted among Men Who have Sex with Men (MSM) attending an MSM clinic in Central Kolkata. The clinic is located in Kadapara area of Central Kolkata and is frequented by MSM and other male clients as well. The clinic runs on Mondays, Wednesdays and Fridays of every week. The objectives of the study were as follows:

1. To find out the different sociodemographic characteristics of the two study groups-Men who have Sex with Men (MSM) and other male clients attending the STD clinic and
2. To compare the physical, mental and social health status of the two study groups.

All the MSM and other male clients visiting the STD clinic during the study period of six months during 2007 were interviewed using Goldberg's 28 point General Health Questionnaire (GHQ).(3) The questionnaire is a self-reporting screening questionnaire, which identifies high probability of suffering from psychiatric illness. It has been well validated and correlates well with consultant psychiatrists. The scoring distinguishes chronic stable complaints from recent exacerbations; an item being counted if the patient thinks that there has been a change from his/her usual self over the last week. The GHQ is used to detect psychiatric disorder in the general population and within community or non-psychiatric clinical settings such as primary care or general medical outpatients. The GHQ-28 is the most well-known and popular version of the GHQ. It contains 28 items that, through factor analysis, have been divided into four subscales. This ‘scaled’ version of the GHQ has been developed on the basis of the results of principal components analysis. The four subscales, each containing seven items, are as follows:

- A - somatic symptoms (items 1-7)
- B - anxiety/insomnia (items 8-14)
- C - social dysfunction (items 15-21)
- D - severe depression (items 22-28)

All items have a 4-point Likert scoring system (0-1-2-3) that ranges from a ‘better/healthier than normal option (allotted score 0)’, through a ‘same as usual (allotted score 1)’ and a ‘worse/more than usual (allotted score 2)’ to a ‘much worse/more than usual (allotted score 3)’ option. The exact wording will depend upon the particular nature of the item. Accordingly, higher the score, the more severe the condition. The threshold score above which psychiatric morbidity is likely is 24 out of a maximum possible score of 84.(3) A total of 108 MSM and 96 other male clients were interviewed by Goldberg's 28-point GHQ during the study period. The questionnaire was translated to local language i.e. Bengali and retranslated back by experts. Interview was conducted after informed consent and on the basis of anonymity. The results were analysed at the end of the study using suitable statistical tests.

## Results and Analysis

Table 1 depicts the sociodemographic features of the two groups. The study population comprised of 108 MSM and 96 other male clients who visited the clinic during the study period. The two groups did not differ significantly with respect to age, literacy status and per capita monthly income (*P* > 0.05). The mean age of MSM was however lower than the mean age of other male clients (22.8 and 26.3 years, respectively). There were more illiterates among other males as compared to MSM group (25 vs. 14%). However, there was lesser number of married males among MSM as compared to other male clients (*P* < 0.05). It was observed that almost 25% of MSM visiting the STD clinic were students while 22.2% were drivers. On the contrary, 51% of the other male clients were drivers. As expected, the proportion of married among MSM (30.6%) was lesser in contrast to other male clients (46%).

**Table 1:** Comparison of sociodemographic characteristics of the study population

Age	MSM (*n*1 = 108)		other clients (*n*2=96)	
		
	No. (%)	Mean ± SD	No. (%)	Mean ± SD
14-18	27 (25)	16.0 ± 1.5	30 (31.3)	16.6 ± 1.1
19-23	36 (33.3)	19.9 ± 0.9	12 (12.5)	21.3 ± 1.5
24-28	21 (19.4)	25.1 ± 1.6	21 (21.9)	25.1 ± 1.6
29-33	18 (16.7)	30.8 ± 1.2	15 (15.6)	30.0 ± 1.1
34-38	3 (2.8)	36.0 ± 1.4	3 (3.1)	35.0 ± 0
39 and above	3 (2.8)	39.3 ± 0.4	15 (15.6)	44.4 ± 3.4
Total	108 (100)	22.8 ± 6.3	96 (100)	26.3 ± 9.6

	**No.**	**%**	**No.**	**%**

Literacy status				
Illiterate	15	13.9	24	25
Just literate/below primary	21	19.4	15	15.6
Primary school completed	9	8.3	6	6.3
Middle school completed	18	16.7	18	18.8
Secondary completed	27	25	21	21.9
Higher secondary completed	11	10.2	10	10.4
Graduate	4	3.7	2	2.1
Post-graduate and above	3	2.8	-	-
Length of stay				
<2 years	24	22.2	12	12.5
>2 years but not since birth	27	25	19	19.8
Since birth	57	52.8	65	67.7
Occupation				
Unemployed	5	4.6	3	3.1
Unskilled workers	11	10.2	10	10.4
Drivers	24	22.2	41	51
Shop owner	12	11.1	3	3.1
Petty traders	9	8.3	2	2.1
Self employed/professionals	17	15.7	16	16.7
Clerical/salesman	3	2.8	12	12.5
Student	27	25	9	9.4
Marital status				
Unmarried	75	69.4	51	53.1
Married	33	30.6	45	46.9
PCI monthly income (in Rs.)				
<500	28	25.9	39	40.6
501-1000	30	27.8	24	25
1001-1500	18	16.7	15	15.6
1501-2000	10	9.3	4	4.2
2001-2500	13	12.0	12	12.5
>2500	9	8.3	2	2.1

*t* = 1.65, df = 202, *P* > 0.05; Chi sq = 4.94, df = 5, *P* > 0.05; Chi sq = 5.23, df = 2, *P* > 0.05; Chi sq = 9.82, df = 1, *P* < 0.01; Chi sq = 5.73, df = 1, *P* < 0.05; Chi sq = 9.14, df = 5, *P* > 0.05

Table 2 shows how the study population fared with regards to GHQ scores, which is a reflection of the status of their physical, mental and social health. The Likert scale of scoring (0-1-2-3) was used in GHQ-28. In each of the four subscales, i.e. somatic symptoms, anxiety and insomnia, social dysfunction and severe depression-the maximum attainable score is 21 while the minimum possible score is 0. Higher the score, worse is the condition.

**Table 2:** Distribution of study population according to general health questionnaire scoring pattern

	MSM (*n*_1_ = 108)		Other clients (*n*_2_ = 96)	
		
	No. (%)	Mean ± SD	No. (%)	Mean ± SD
Somatic symptoms scoring				
0-6	21 (19.4)	4.4 ± 1.6	15 (15.6)	3.0 ± 1.7
7-13	78 (72.2)	10.1 ± 2.2	51 (53.1)	10.4 ± 1.7
14-21	9 (8.3)	16.0 ± 0.9	30 (31.3)	16.2 ± 1.5
Total	108 (100)	9.5 ± 3.6	96 (100)	11.0 ± 4.6
	Secured minimum score = 2, max. score = 16		Secured minimum score = 1, max. score = 19	
Anxiety and insomnia scoring				
0-6	42 (38.9)	5.3 ± 0.8	51 (53.1)	3.4 ± 1.8
7-13	42 (38.9)	10.6 ± 1.63	33 (34.4)	9.0 ± 1.2
14-21	24 (22.2)	15.7 ± 1.5	12 (12.5)	16.8 ± 0.8
Total	108 (100)	9.7 ± 4.2	96 (100)	7.0 ± 4.8
	Secured minimum score = 4, max. score = 14		Secured minimum score = 0, max. score = 18	
Social dysfunction scoring				
0-6	33 (30.6)	5.5 ± 0.7	42 (43.7)	2.3 ± 1.8
7-13	48 (44.4)	9.7 ± 1.9	39 (40.6)	10.4 ± 1.9
14-21	27 (25.0)	15.1 ± 1.3	15 (15.7)	15.8 ± 1.2
Total	108 (100)	9.8 ± 3.8	96 (100)	7.6 ± 5.6
	Secured minimum score = 4, max. score = 17		Secured minimum score = 0, max. score = 18	
Severe depression scoring				
0-6	81 (41.7)	2.6 ± 1.9	78 (65.6)	2.0 ± 1.6
7-13	21 (44.4)	10.3 ± 2.0	15 (25)	8.6 ± 1.4
14-21	6 (13.9)	15.50 ± 0.5	3 (9.4)	19 ± 0.0
Total	108 (100)	4.8 ± 4.4	96 (100)	3.6 ± 4.0
	Secured minimum score = 0, max. score = 16		Secured minimum score = 0, max. score = 14	
Net GHQ score				
≤24	39 (36.1)	19.0 ± 2.2	44 (45.8)	18.5 ± 3.1
>24	69 (63.9)	42.0 ± 9.3	52 (44.2)	36.5 ± 9.8
Total	108 (100)	33.8 ± 13.3	96 (100)	29.2 ± 11.8
Minimum possible score = 0,	Secured minimum score = 15, max. score = 65		Secured minimum score = 12, max. score = 62	
Maximum possible score = 84				

*t* critical two-tail value = 1.97, df = 202, *P* < 0.01; *t* critical two-tail value = 1.97, df = 202, *P* < 0.01; *t* critical two-tail value = 1.97, df = 202, *P* < 0.01; *t* critical two-tail value = 1.97, df = 202, *P* < 0.05; *t* critical two-tail value = 1.97, df = 202, *P* < 0.05

As regards to somatic symptoms, the highest score obtained by a MSM was 16 while the same for other male clients was 19. Somatic symptoms were found to be significantly more (*P* < 0.05) among other male clients as compared to MSM (the mean scores were 11.0 and 9.5, respectively). More non-MSM clients as compared to MSM had a ‘more than usual feeling’ that they are ‘getting pains in their head’ (20 vs. 16%), ‘feeling run down and out of sorts’ (42 vs. 22%) and their ‘health was worse than usual (56 vs. 30%). Anxiety and insomnia were elicited in the study population through a set of seven questions as per GHQ-28. The scores of MSM regarding this aspect ranged from 4 to 14 while other male clients scored between 0 and 18 in the same set of questions. However, it was observed that more MSM were suffering from anxiety and insomnia as compared to other male clients (mean score 9.7 ± 4.2 vs. 7.0 ± 4.8) and the difference was found significant (*P* < 0.01). It was observed that more MSM as compared to other male clients had a ‘more than usual feeling’ that they had ‘lost much sleep over worry’, (44 vs. 39%), ‘been getting edgy and bad tempered’ (64 vs. 20%) and ‘feeling nervous and strung up all the time’ (69 vs. 38%). Social disharmony and discord was found to be significantly more prevalent among MSM than other male clients (mean score 9.8 ± 3.8 vs. 7.6 ± 5.6). Scores in this aspect ranged from 4 to 18 among MSM, while same among other male clients was 0 to 18. It was found that more MSM in comparison to other males had been taking ‘longer than usual to get things done’ (21 vs. 14%), feeling ‘less than satisfied with the way they are carrying out their task’ (28 vs. 26%) and been able to ‘enjoy day-to-day activities much less than usual’ (42 vs. 21%). The MSM were found to be suffering more from severe depression (4.8 ± 4.4) as compared to other male clients (3.6 ± 4.0) and this association was found to be statistically significant (*P* < 0.05). The MSM scored between 0 and 16 on questions eliciting severe depression while the same for other male clients was 0-14. However, more MSM as compared to other male clients had a ‘more than usual feeling’ of thinking themselves as ‘worthless’ (12 vs. 6%), feeing ‘life is hopeless’ (10 vs. 9%) and ‘wishing he was dead and away from it all’ (9 vs. 5%). Overall, the mean GHQ score of MSM (33.8 ± 13.3) was significantly higher (*P* < 0.05) than the mean score of the other male clients (29.2 ± 12.8). Using the recommended threshold score of >24 (threshold being defined as ‘just significant clinical disturbance’ or that point where probability of being a ‘case’ suffering from psychiatric morbidity is 50%), significantly more MSM (63.9%) were found to have attained the score of > 24 as compared to other male clients (44.2%) [*P* < 0.05].

## Discussion

Males are often easier to access for sex than females, while male sex workers are usually cheaper than female sex workers are. Men Who have Sex with Men includes self-identified gay men, primarily among the urban, English-speaking elite and middle classes. At the same time, MSM includes day-to-day causal laborer and the next-door child laborer working in a tea stall or a sweet shop. Without a welfare system, and with significant levels of unemployment or low-level incomes, male sex work can be a way out in terms of supporting the self and family. This is not to imply that males involved in sex work do not enjoy the sex with other males. Often they may also have a regular male partner, and/or a wife or girlfriend. Thus what can be said to exist in India are a range of masculinities and genders with differing contextualization of sexual behaviors, sex partner choices, perceived sexual needs, pleasures and desires, where male-to-male sex is seen primarily within a gendered dynamic, rather than in terms of sexual orientation or identity.(4) There is definitely an insensitive attitude towards this population leading to their social exclusion and deprivation of service provision, treatment and care. More often than not, they suffer from problems related to mental health. Sadly, this group cannot open up their feelings to their friends and closed ones; as a result they suffer from mental stress and morbidities pertaining to mental health apart from a host of physical ailments.

This is clearly depicted in the study by the researchers where more than half (63.9%) of the MSM crossed the threshold score of 24 signaling a high risk of psychiatric illness in the group. This, despite the fact that they were already suffering from physical morbidities for which they visited the clinic, speaks volumes about the sordid state of mental health of MSM. It is worth mentioning that significantly more number of MSM crossed the threshold GHQ score of 24 as compared to other male clients (*P* < 0.05) who also visited the clinic with a host of physical morbidities. The proportion of other male clients who crossed the threshold score was also fairly high (44.2%). Perhaps their physical morbidities took a toll on their mental health as well. A study by Wright *et al*. in the Department of Medicine, University of Dundee, UK(5) on 234 patients using GHQ-28 showed that 29% of the women and 25% of the men crossed the threshold score. Another study by Swallow *et al*. in the antenatal clinic in University of Lincoln, UK(6) using similar GHQ-28 questionnaire revealed that 40% of the pregnant women were likely to be suffering from psychiatric morbidities after having crossed the threshold score of 24.

## Conclusion

It is needless to say that awareness towards prevention of HIV/AIDS and other STD need to be increased among MSM and other clients visiting the MSM clinic in various parts of the country. But the researchers also felt the need of provision of counseling and behavior therapy by trained psychiatrists in such clinics especially since a huge proportion of clients are suffering from varied psychiatric morbidities. Alternatively, medical officers who man these clinics need to be given special training in treatment of psychiatric morbidities or trained psychologists need to be posted in such clinics with separate room for counseling sessions. This will definitely help to improve the overall mental and social health of the clients along with their physical health as we march towards the idealistic definition of positive health in the years to come.
